# Integrated transcriptomic and WGCNA analyses reveal candidate genes regulating mainly flavonoid biosynthesis in *Litsea coreana* var. *sinensis*

**DOI:** 10.1186/s12870-024-04949-1

**Published:** 2024-04-01

**Authors:** Na Xie, Qiqiang Guo, Huie Li, Gangyi Yuan, Qin Gui, Yang Xiao, Mengyun Liao, Lan Yang

**Affiliations:** 1 Institute for Forest Resources and Environment of Guizhou, College of Forestry, Guizhou University, Guiyang, 550025 China; 2 College of Agriculture, Guizhou University, Guiyang, 550025 China

**Keywords:** *Litsea coreana* var. *sinensis*, Flavonoids, *De novo* transcriptome sequencing, WGCNA, Candidate genes

## Abstract

*Litsea coreana* Levl. var. *sinensis* (Allen) Yang et P. H. Huang is a popular ethnic herb and beverage plant known for its high flavonoid content, which has been linked to a variety of pharmacological benefits and crucial health-promoting impacts in humans. The progress in understanding the molecular mechanisms of flavonoid accumulation in this plant has been hindered due to the deficiency of genomic and transcriptomic resources. We utilized a combination of Illumina and Oxford Nanopore Technology (ONT) sequencing to generate a *de novo* hybrid transcriptome assembly. In total, 126,977 unigenes were characterized, out of which 107,977 were successfully annotated in seven public databases. Within the annotated unigenes, 3,781 were categorized into 58 transcription factor families. Furthermore, we investigated the presence of four valuable flavonoids—quercetin-3-O-*β*-D-galactoside, quercetin-3-O-*β*-D-glucoside, kaempferol-3-O-*β*-D-galactoside, and kaempferol-3-O-*β*-D-glucoside in 98 samples, using high-performance liquid chromatography. A weighted gene co-expression network analysis identified two co-expression modules, MEpink and MEturquoise, that showed strong positive correlation with flavonoid content. Within these modules, four transcription factor genes (*R2R3*-*MYB*, *NAC*, *WD40*, and *ARF*) and four key enzyme-encoding genes (*CHI*, *F3H*, *PAL*, and *C4H*) emerged as potential hub genes. Among them, the *R2R3*-*MYB (LcsMYB123*) as a homologous gene to *AtMYB123/TT2*, was speculated to play a significant role in flavonol biosynthesis based on phylogenetic analysis. Our findings provided a theoretical foundation for further research into the molecular mechanisms of flavonoid biosynthesis. Additionally, The hybrid transcriptome sequences will serve as a valuable molecular resource for the transcriptional annotation of *L. coreana* var. *sinensis*, which will contribute to the improvement of high-flavonoid materials.

**Supplementary Information:**

The online version contains supplementary material available at 10.1186/s12870-024-04949-1.

## Introduction

Tea is a popular non-alcoholic beverage worldwide. *Litsea coreana* Levl. var. *sinensis* (Allen) Yang et P. H. Huang, a member of the Lauraceae family, serves as the primary botanical source for Eagle tea production [1]. For centuries, Eagle tea has been consumption in southern China both as a popular health beverage and as a traditional hypolipidemic medication. It has exhibited diverse biological and pharmaceutical properties such as hypoglycemic inhibition, lipid-lowering effects, immune enhancement, antioxidant activity, nitrite scavenging ability, and nitrosamine formation inhibition [2]. These attributes can be primarily ascribed to the substantial presence of flavonoids within this botanical species. As the main active ingredient in *L. coreana* var. *sinensis*, flavonoids can be divided into flavonols, anthocyanins, chalcones, dihydroflavonols, isoflavones, flavones, and flavanols depending on the carbon of the C-ring to which the B-ring is linked as well as the level of unsaturation and oxidation of the C-ring [3]. Flavonols have various functions, including attracting insects for pollination, communicating with microbes, protecting against UV radiation, controlling plant growth, and affecting pollen fertility [4]. In tea leaves, flavonols are mainly found as O-glycosides with a glycoside moiety located at the C-3 position of aglycones such as quercetin, kaempferol, and myricetin [5]. These flavonol glycosides are not only associated with tea flavor, but also have an influence on its coloration [6, 7]. It has been identified that *L. coreana* var. *sinensis* chiefly comprises four variants of flavonol glycosides [8], namely quercetin-3-O-*β*-D-galactoside (Q-3-gal), quercetin-3-O-*β*-D-glucoside (Q-3-glu), kaempferol-3-O-*β*-D-galactoside (K-3-gal) and kaempferol-3-O-*β*-D-glucoside (K-3-glu) [9]. Several studies have confirmed that Q-3-gal, Q-3-glu, K-3-gal, and Q-3-glu have a variety of biological activities, including anti-inflammatory [10], antioxidant [11], antidepressant [12], and antimicrobial effects [13]. For example, K-3-gal has been proved to have significant procoagulant activity in vitro [14]. K-3-glu enhances TNFα-induced cell death by blocking NF-кB activity and effectively inhibits tumor growth while inducing cancer cell apoptosis [15]. Q-3-gal triggers apoptosis in breast cancer cells through the ROS-mediated NF-κB signaling pathway [16]. Q-3glu offers potential as a therapeutic intervention for neurodegenerative diseases [17]. In light of its noteworthy medicinal and nutritional attributes, the synthesis of flavonoids and the corresponding molecular mechanisms have become an area of great interest among scholars. Nevertheless, the biosynthetic pathway and regulatory genes in *L. coreana* var. *sinensis* remains unclear.

Flavonoid biosynthesis is an intricate process involving both early and late biosynthetic genes [18]. Most of the genes encoding enzymes involved in flavonoid biosynthesis have been discovered in plants [19]. Flavonoids are initially synthesized catalyzed by phenylalanine ammonia-lyase (PAL), followed by cinnamate 4-hydroxylase (C4H), 4-coumarate: CoA ligase (4CL), chalcone synthase (CHS), chalcone isomerase (CHI), flavanone 3-hydroxylase (F3H), flavonoid 3’-hydroxylase (F3’H), flavonol synthase (FLS), and UDP-glucosyltransferase (UGT) [20]. Transcription factors (TFs) such as bZIP, WRKY, MADS-box, ARF, NAC, and especially bHLH, MYB, and WD40 controlled flavonoid biosynthesis through regulating the biosynthetic genes involved in flavonoid biosynthesis pathway [21–23]. Among these, bHLH TFs can control anthocyanin, PA, and flavonol biosynthesis within the flavonoid pathway [24]. A key player in the regulatory networks that govern flavonoid biosynthesis in plants is the R2R3-MYB TFs. In *Arabidopsis thaliana*, there are 126 R2R3-MYB TFs, which can be divided into 25 subgroups, and the 4th, 5th, 6th, 7th, 15th, 19th subgroups are directly implicated in the biosynthesis of flavonoids [25–27]. For instance, the SG7 MYBs (*MYB11/PFG2, MYB12/PFG1 and MYB111/PFG3*) and SG19 MYBs *(MYB21, MYB24*, and *MYB57*) control flavonol biosynthesis [23, 28]. Furthermore, *MYB123/TT2*, a member of subgroup 5, is involved in the biosynthesis of proanthocyanidins (PAs) [29], while *AtMYB75/PAP1*, *AtMYB90/PAP2*, *AtMYB113* and *AtMYB114*, all of subgroup 6, are known to modulate anthocyanin biosynthesis [30]. In addition, MYB TFs can also interact with bHLH and WDR proteins to form a dynamic transcriptional activation complex (MBW complex) to regulate the anthocyanin and PA biosynthesis [31].

The assembly and annotation of a high-grade, chromosome-scale genome of *L. coreana* var. *sinensis* was recently accomplished, yet the resulting data remains unpublished [32]. RNA-seq based on high-throughput next-generation sequencing has been widely applied in plant studies, such as *Pinus massoniana* [33], and *Camellia sinensis* [34]. The majority of transcriptomes are currently created utilizing second-generation sequencing (SGS) technologies on the Illumina platform [35]. However, Illumina reads’ short length limits their accurate identification of complex transcript isoforms [36]. The third-generation sequencing (TGS) technologies, such as Pacific Bioscience (PacBio) and Oxford Nanopore Technologies (ONT), have the capability to process substantial data quantities and read long sequences and full-length gene transcripts [37]. Compared to PacBio, ONT provides full-length, single-molecule transcriptome analysis, with exceptionally long reads, at a reduced cost and with a more straightforward operation [38]. The integration of second- and third-generation sequencing techniques can produce high-quality assemblies that facilitate the investigation of complex biological questions [39].

Weighted gene co-expression network analysis (WGCNA) is a valuable R tool that integrates gene expression data with phenotypic information to identify key modules and hub genes associated with target traits. This approach effectively clusters genes exhibiting similar expression patterns into distinct modules, which are characterized by shared functions or pathways [40]. By leveraging plant life activities and high-throughput sequencing technology, WGCNA enables the acquisition of multiple expression traits and facilitates the construction of a scale-free network topology centered around pivotal regulatory genes [41]. Hou et al. combined WGCNA and transcriptomic analyses revealed a co-expression gene network associated with rutin synthesis [42]. Through the application of WGCNA, MYB, ERF, WRKY, bZIP, and bHLH were identified in mango for their role in flavonoid accumulation (*Mangifera indica*) [43]. A concurrent study by Ju et al. [44] identified 17 co-expression modules and found MEcoral1 module was related to flavonoid metabolism.

Flavonoids are the main active ingredients in *L. coreana* var. *sinensis*; however, the molecular mechanisms underlying the characteristic accumulation of flavonoids are largely unknown. Transcriptome libraries greatly improved transcriptome sequencing data of *L. coreana* var. *sinensis*, providing valuable insights for future studies on flavonoid biosynthesis genes. WGCNA analysis identified potential hub genes for flavonoid production, offering candidate genes for industrial development and genetic improvement of *L. cor*eana var. *sinensis* in agriculture and biotechnology research fields.

## Materials and methods

### Plant materials

The fresh tea shoots with one bud and two leaves were collected from 98 samples of *L. coreana* var. *sinensis* (22 years old) from five regions: (1) Dao Zhen county (29° 09′ N, 107° 29′ E, *n* = 20) (2) Xi Shui county (28° 31′ N, 106° 29′ E, *n* = 17) (3) Kai Yang county (27° 10′ N, 107° 05′ E, *n* = 23) (4) Mei Tan county (27° 43′ N, 107° 27′ E, *n* = 23) (5) Zhen An county (28° 25′ N, 107° 26′ E, *n* = 15), Guizhou Province, China (Table S3). The altitude varies between 600 m and 900 m, while the average annual temperature fluctuates within the range of 13.2 ℃ to 15.6 ℃. Moreover, the average annual precipitation spans from 1080 mm to 1255 mm. The climate exhibits mild and humid characteristics, influenced by continental factors from the monsoon, while the soil predominantly consists of purple shale-derived soil [1]. The identification process was conducted by Dr. Zhi Li to ensure the accuracy and reliability of the samples. A scrupulous selection process was applied to ensure the health and uniformity of the samples in terms of height and canopy width. The voucher specimen has been deposited in the Herbarium, College of Forestry, Guizhou University, Guiyang, China (voucher specimen: GZAC0028542). All samples were immediately frozen in liquid nitrogen and stored at -80 °C before RNA extraction and metabolite analysis.

### Flavonoid quantification

The extraction and determination of flavonoids were performed using a previously published method with slight modifications [9, 45]. The standards of flavonoids (K-3-glu, Q-3-glu, K-3-gal, and Q-3-gal) used to create a calibration curve were purchased from Shanghai Yuanye Bio-Technology Co., Ltd (Shanghai, China). Quantitative analysis was carried out based on the calibration curves of four standards. Flavonoids were quantified using a Shimadzu LC-20 A HPLC system, equipped with Shim-pack GIST C18 (250 × 4.6 mm, 5 μm, Shimadzu, Japan). The water used was prepared with a Millipore water purification system. The freeze-dried samples were pulverized for 30 s at 30 Hz. Each sample, weighing 0.5 g (dry weight), was carefully weighed and subsequently mixed with 20 ml of ethanol: water (7:3, v/v). This mixture was subjected to an extraction process for a duration of 60 min, and then sonicated at a frequency of 40 kHz for an additional 20 min. Following centrifugation at 5000 rpm for 15 min, the supernatants were diluted and subsequently filtered through a microporous filter membrane (0.22 μm pore size) before being transferred into HPLC vials. The final concentration of the extract analyzed had a final concentration of 4 mg/ml. The mobile phase was composed of aqueous acetic acid solution (1:500, v/v) (A) and acetonitrile (B), and the elution program was as follows: a linear gradient from 16 to 24% B for 0–26 min, and then 20% B for 26–40 min, followed by washing and equilibration. The injection volume was 10 µl with a flow rate of 1 ml/min and detected at a wavelength of 355 nm, while column temperature was kept at 35℃.

### RNA extraction, library construction, and sequencing

The RNA-prep Pure Plant Plus Kit (TIANGEN, Beijing, China) was used to extract total RNA from 98 samples. The integrity and purity of the isolated RNA were determined using a NanoPhotometer spectrophotometer (Thermo Scientific, Waltham, USA) and an Agilent 2100 Bioanalyzer (Agilent Technologies, California, USA), respectively. RNA was reverse transcribed into cDNA using U-mRNAseq Library Prep Kit (AT4221). The synthesized cDNA was purified using magnetic beads, the ends were repaired, and a single nucleotide A was added. Sequencing adaptors were ligated onto each blunt-ended cDNA molecule. The prepared samples were subsequently sequenced using an Illumina NovaSeq 6000 platform, resulting in the generation of paired-end reads. The raw reads were filtered by removing the contamination adapter reads, low-quality reads, and those containing more than 5% “N” reads using fastp (v0.21.0).

Oxford Nanopore Technologies’ standard cDNA library sequencing protocol was used for preparing the Nanopore sequencing libraries from captured RNA. Two samples were randomly selected from each of the five places, resulting in a total of 10 samples being mixed equally for the library construction. cDNA was synthesised using the DNA-PCR Sequencing Kit (SQK-PCS11). The prepared library was sequenced on the Oxford Nanopore PromethION platform. The Illumina short reads and Oxford Nanopore long reads were subjected to hybrid assembly using the rnaSPAdes (v3.15.2) [46]. The completeness of transcriptome was assessed by the BUSCO method with the embryophyta _odb10 dataset [47]. The clean reads were aligned to the assembled transcripts with Bowtie 2 (v2.4.2). Expression levels were measured using transcripts per kilobase of per million mapped reads (TPM) (Kallisto v0.46.2). TransDecoder (v3.0.0) (https://github.com/TransDecoder/TransDecoder/wiki) was used for gene prediction. The entire process of library construction and transcriptome sequencing were carried out by Kaitai-bio-Company (Hangzhou, China).

### Gene functional annotation and transcription factor identification

The functional annotations of the unigenes were conducted through BLASTx, utilizing an E-value threshold of 10^− 5^ across seven public databases: non-redundant protein sequences (NR), eukaryotic Orthologous Groups (KOG), the database of Homologous protein family (Pfam), non-redundant protein sequence database (Swiss-Prot), Translation of EMBL (TrEMBL), Gene Ontology (GO) and Kyoto Encyclopedia of Genes and Genomes (KEGG). NR database results were preferentially selected. The identification of transcription factor families was achieved by querying the unigenes against the transcription factor protein sequences contained in the Plant Transcription Factor Database (PlantTFDB; http://planttfdb.gao-lab.org//v5.0).

### Weighted gene co-expression network analysis

The WGCNA was performed using the WGCNA R package (v4.1.3) [48]. The log_10_(TPM + 1) normalization method was applied to the TPM expression matrix, with a data filter that excluded genes having a log_10_(TPM + 1) value below 1 in over 30% of the samples [49]. Median absolute deviation (MAD) was used to select the top 20,000 genes focusing on those displaying large inter-sample variation for constructing a co-expression network. Genes demonstrating high degrees of clustering were allocated to distinct modules. The expression profile of module genes in each sample was displayed by module eigengene, which is the first principal component of gene expression. Gene modules were calculated based on the default settings with minmodulesize = 30, mergecutheight = 0.25, and power = 6 (Fig. S4). The correlations between modules and four flavonoids content were determined by estimating the module-trait relationships and KEGG enrichment analysis was performed on all modules using the Omicshare platform (https://www.omicshare.com/) [38]. The resulting networks were then visualized, and the degree value was calculated using Cytoscape (v3.10.0). In the co-expression module, hub genes were predominantly employed to represent closely related genes exhibiting substantial connectivity. The top 30 genes in each module based on degree were identified as hub genes. From the target modules, we pinpointed the transcription factor genes involved in the flavonoid biosynthesis pathways, in addition to the structural genes. TBtools (v1.120) was utilized to generate gene expression heatmaps for selected samples and candidate genes [50].

### Phylogenetic analysis

The R2R3-MYB protein sequences related to flavonoid biosynthesis from different plants were obtained from the NCBI database (http://www.ncbi.nlm.nih.gov/). The sequences were aligned using ClustalW and the phylogenetic tree was generated with 1000 bootstrap replicates using the neighbor-joining method with p-distance model in MEGA7.0 [51], then visualized by the iTOL web tool (https://itol.embl.de/). Sequences were visualized using GeneDoc. Accession numbers: AtMYB113 (AT1G66370.1), AtPAP1 (AAG42001), AtMYB114 (AT1G66380.1), VvMYBA1 (BAD18977), MdMYB10 (ACQ45201.1), PbMYB10b (KT601122), PbMYB114 (MF489219), MdMYB9 (ABB84757.1), AtMYB11 (NP_191820), AtMYB12 (NC_003071.7), AtMYB111 (AAK97396), VvMYBF1 (NP_001267930.1), MdMYB22 (AAZ20438.1), AtMYB60 (AT1G08810.1), AtMYB32 (AT4G34990.1), AtMYB3 (AEE30263.1), AtMYB4 (AT4G38620), VvMYB4a (ABL61515.1), MdMYB16 (HM122617).

### qRT-PCR analysis

To validate the accuracy and reliability of the RNA-seq data, 10 genes related to the flavonoid biosynthesis pathway from pink module were selected for validation using qRT-PCR. Sample XS13 was randomly selected for fluorescence verification. Total RNA was extracted using RNA-prep Pure Plant Plus Kit. Reverse transcription was performed using TUREscript RT Kit with gDNA Eraser (Aidlab, Beijing, China) and real-time PCR conducted using 2×Sybr Green qPCR Mix (Aidlab, Beijing, China) on An IQ5 real-time PCR detection system (Bio-Rad) (Applied Biosystems, Waltham, USA). The 18 S rRNA gene was utilized as the internal control. The qPCR was performed as follows: 94 °C for 2 min, 40 cycles of 94 °C for 5 s, 60 °C for 30 s. The relative expression levels were calculated based on the values of three technical replicates and the 2 ^−ΔΔCt^ method was used for expression calculations [52]. Primers utilized for qRT-PCR procedure were designed by Primer 3 (v0.4.0) (http://primer3.ut.ee) and listed in Table S1. The primers were designed to amplify a 100–300 bp product with a melting temperature (Tm) of around 60 °C and an approximate GC% content of 50%. Data were analyzed by Excel 2010.

### Statistical analysis

Data were presented as mean value ± standard error (SE). The differences among four flavonoid contents and flavonoid contents in different regions were evaluated via a one-way analysis of variance (ANOVA) and significant differences were calculated using the least significant difference (LSD) test (defined as *P* < 0.05). All statistical analysis were performed using SPSS software (version 19), and all the figures were plotted by Origin 2018 software.

## Results

### Determination of the main flavonoid contents

**Fig. 1:**
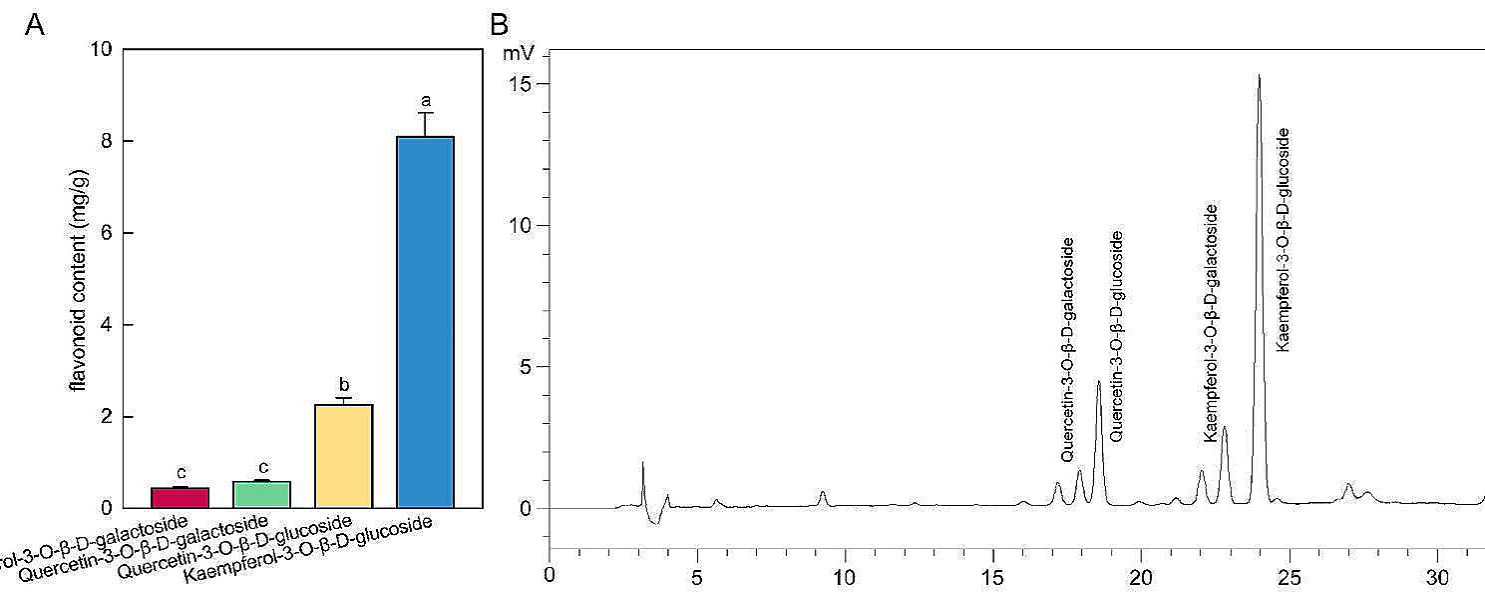
The main flavonoid content analysis of *L. coreana* var. *sinensis.* (A) Flavonoid contents assessed by HPLC fingerprint analysis (mg/g dry weight). Different letters indicate significant differences (*P* < 0.05). (B) Typical HPLC chromatograms of *L. coreana* var. *sinensis*

Flavonoids, especially flavonols, are potentially health-promoting components in the human diet. HPLC analysis was performed on 98 samples to compare the levels of major flavonoids and explore regional variations. The results showed significant differences among these four kinds of flavonol glycosides, as well as in samples collected from different regions. The most prevalent flavonoid within the samples was K-3-glu, which exhibited the the highest content, followed by Q-3-glu, Q-3-gal and K-3-gal (Fig. 1A and B). The average contents of K-3-glu and Q-3-glu were determined to be 8.00 and 2.26 mg/g, respectively. In comparison, the content of K-3-glu was much higher than other components. The average content of K-3-glu in DZ, KY, MT, ZA, XS were 2.36, 8.79, 10.23, 6.40, 11.95 mg/g, respectively. Moreover, the average contents of K-3-glu, K-3-gal, Q-3-glu and Q-3-gal in XS were 11.95, 0.54, 2.91 and 0.64 mg/g, respectively (Table 1), while these values in DZ were only 2.36, 0.18, 0.70, 0.21 mg/g, respectively.

**Table 1:** The main flavonoid contents in *L. coreana* var. *sinensis* from different regions (mg/g)

flavonoid	DZ	KY	MT	ZA	XS
Kaempferol-3-O-β-D-glucoside	2.36 ± 0.26^d^	8.79 ± 0.75^bc^	10.23 ± 1.07^ab^	6.40 ± 0.86^c^	11.95 ± 1.34^a^
Kaempferol-3-O-β-D-galactoside	0.18 ± 0.01^c^	0.48 ± 0.04^ab^	0.52 ± 0.05^ab^	0.37 ± 0.04^b^	0.54 ± 0.06^a^
Quercetin-3-O-β-D-glucoside	0.70 ± 0.09^c^	2.41 ± 0.19^ab^	3.19 ± 0.35^a^	1.92 ± 0.23^b^	2.91 ± 0.46^a^
Quercetin-3-O-β-D-galactoside	0.21 ± 0.02^c^	0.61 ± 0.06^b^	0.86 ± 0.09^a^	0.54 ± 0.07^b^	0.64 ± 0.10^b^
Total	3.45	11.68	14.8	9.23	16.04

Different letters in the column indicate significant differences in the origin of each compound (*P* < 0.05)

### Transcriptome sequencing and *de novo* assembly

Individual transcriptome assemblies were generated for each of the 98 samples. Approximately 50,267,369 raw reads were generated using an Illumina sequencing platform. The Q20 (sequencing error rate < 1%), Q30 (sequencing error rate < 0.1%), and GC content among all the libraries were about 98.05%, 95.05%, and 47.58% (Table 2). After raw reads filtered, 37.28–71.54 million clean reads were obtained, with 5.54–10.65 G clean base. The full-length transcriptome sequencing yielded 10,566,379 clean reads with an average length of 1199.9 bp and an N50 length of 1,345 bp. The transcriptome assembled by rnaSPAdes has a contig N50 of 2,494 bp and a GC content percentage of 40.29%. The distribution of the transcripts by size was as follows: 61.55% were between 201 and 1000 bp in length, 27.06% were between 1001 and 3000 bp, and the remaining 11.39% exceeded 3000 bp in length (Fig. S1). BUSCO analysis revealed that the transcriptome had 96.3% complete (22.1% as single-copy and 74.2% as duplicated), 3.2% fragmented and 0.5% missing BUSCOs. A total of 167,077 unigenes were predicted using TransDecoder. After extracting the longest transcriptome, 126,977 unigenes remained. Additionally, 107,977 unigenes were successfully annotated in the public databases.

**Table 2:** Quality of reads obtained after Illumina-sequencing

Classification	Maximum	Minimum	Average
Raw Reads	72,776,102	37,886,518	50,267,369
Clean Reads	71,541,570	37,282,590	49,247,699
Clean Bases (G)	10.65	5.54	7.33
Q20 (%)	98.50	96.84	98.05
Q30 (%)	95.88	93.43	95.05
GC Content (%)	49.36	46.37	47.58

### Functional annotation and transcription factor analysis

**Fig. 2:**
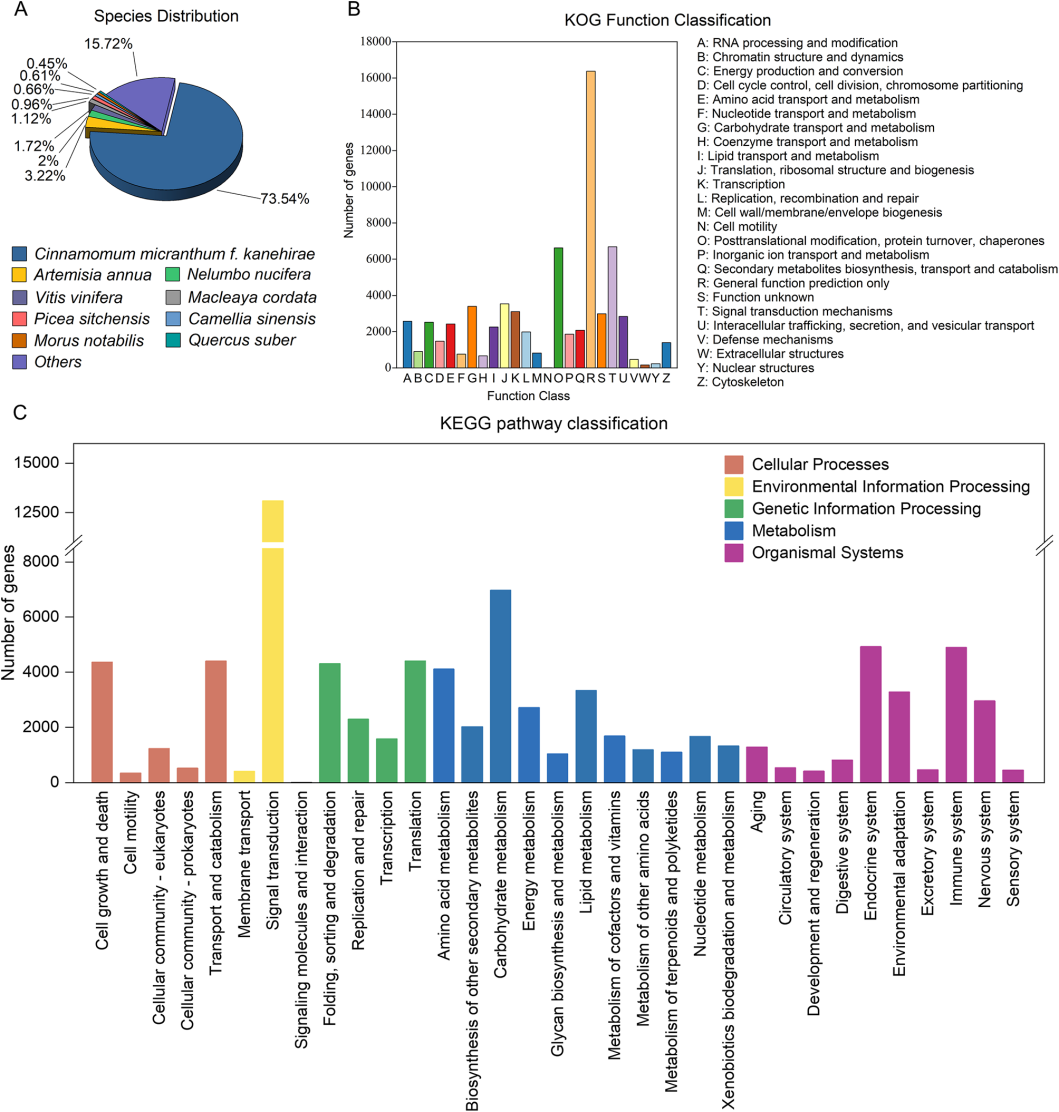
Gene annotation and functional classification. (**A**) Homologous species distribution annotated in the NR database. (**B**) The KOG categories of the unigenes. (**C**) Annotation in KEGG pathway classification. The image was created by the author, and KEGG copyright permission was obtained

To obtain comprehensive and extensive annotation information, we performed sequence annotation by conducting similarity searches across seven databases, including GO, KEGG, KOG, NR, Pfam, Swiss-Prot, and TrEMBL. Specifically, 52,892 (41.65%), 34,899 (27.48%), 61,557 (48.48%), 76,446 (60.20%), 96,438 (75.95%), 66,072 (52.03%), and 53,978 (42.51%) unigenes were annotated respectively (Table S2). Similarity analysis comparison of all predicted proteins found that majority of them are homologous to the species *Cinnamomum micranthum f. kanehirae* (73.54%), followed by *Artemisia annua* (3.22%), *Nelumbo nucifera* (2%), *Vitis vinifera* (1.72%), *Macleaya cordata* (1.12%), *Picea sitchensis* (0.96%), and *Camellia sinensis* (0.66%), among others (Fig. 2A).

Based on sequence similarity analysis, a total of 52,892 unigenes were annotated to one or more GO terms, and classified into three primary functional categories: biological process (BP), cellular component (CC), and molecular function (MF) (Fig. S2). The BP category was dominated by the terms ‘cellular process’ (35,412 unigenes) and ‘metabolic process’ (28,503 unigenes). The MF category featured ‘catalytic activity’ (24,745 unigenes), ‘binding’ (19,144 unigenes), and ‘transcription regulator activity’ (3,692 unigenes) as the top subcategories. For the CC category, the leading terms were ‘cell part’ (46,078 unigenes), ‘cell’ (46,078 unigenes), ‘organelle’ (35,713 unigenes), ‘organelle part’ (19,515 unigenes) and ‘membrane’ (18,020 unigenes). The KOG analysis classified 61,557 unigenes into 25 different categories (Fig. 2B). The most common subclasses were ‘general function prediction only’ (16,375 unigenes), ‘signal transduction mechanisms’ (6,684 unigenes) and ‘posttranslational modification, protein turnover, chaperones’ (6,624 unigenes). Totally, 34,899 unigenes annotated in the KEGG database, of which ‘signal transduction’ (13,084 unigenes), ‘carbohydrate metabolism’ (6,968 unigenes) and ‘endocrine system’ (4,921 unigenes) were the top three pathways in the *L. coreana* var. *sinensis* (Fig. 2C).

TFs are integral regulators of plant growth and metabolism, functioning as both activators and repressors. A total of 3,781 unigenes could be further classified into 58 TF families. Among these, bHLH transcription factors were the most abundant (314, 8.30%), succeeded by NAC (253, 6.69%), MYB-related (240, 6.35%), C2H2 (219, 5.72%), bZIP (192, 5.08%), ERF (188, 4.97%), and MYB (180, 4.76%) (Fig. S3). This comprehensive identification of TFs offers an abundant source of information for studying the roles of TFs in various biochemical pathways in *L. coreana* var. *sinensis.*

### Weighted gene co-expression network analysis

**Fig. 3:**
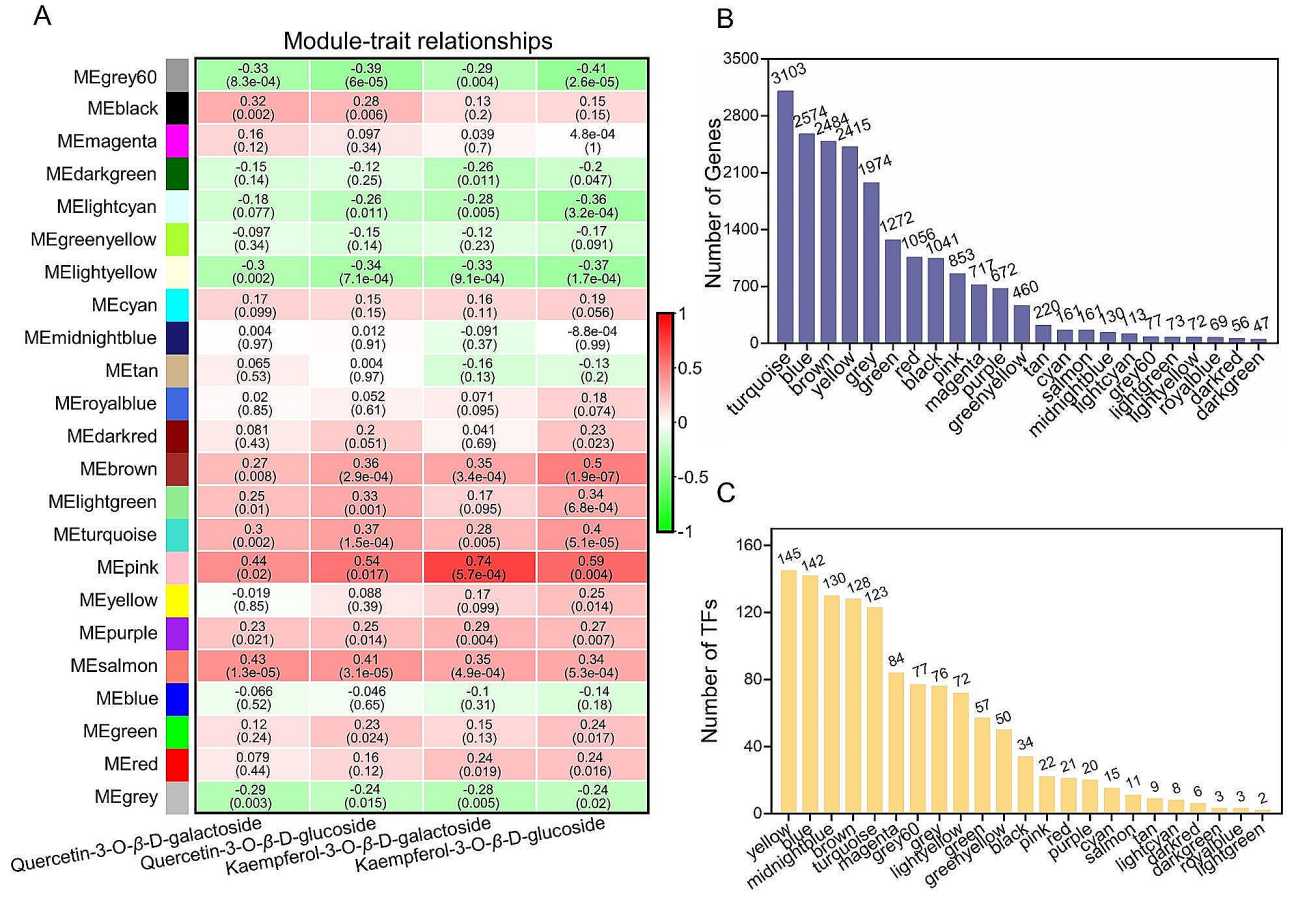
The results of WGCNA. (**A**) Correlation coefficient between flavonoids and module eigengenes with red and blue representing positive and negative correlations, respectively. Each column corresponds to K-3-gal, Q-3-glu, Q-3-gal and K-3-glu values, and each row represents a module. *p*-values are shown in parentheses. The x-axis represents the four flavonoids, while the y-axis represents various modules. (**B**) The number of genes in each module. (**C**) The number of TFs in each module

To identify candidate genes related to flavonoid biosynthesis, we conducted a WGCNA based on gene expression data and the contents of four flavonoids. As a result, 23 modules were obtained, including the grey module, which contained all genes that did not belong to any other modules (Fig. 3A). The size of modules ranged from 47 to 3103 unigenes (Fig. 3B). TFs present in each module varied in quantity from 2 to 145 (Fig. 3C). In addition, the relationship between modules and flavonoids were examined and the pink module displayed a significantly strong positive correlation with four flavonoids, with the highest correlation observed with K-3-gal (*r* = 0.74, *p* = 5.7e*10^− 4^).

**Fig. 4:**
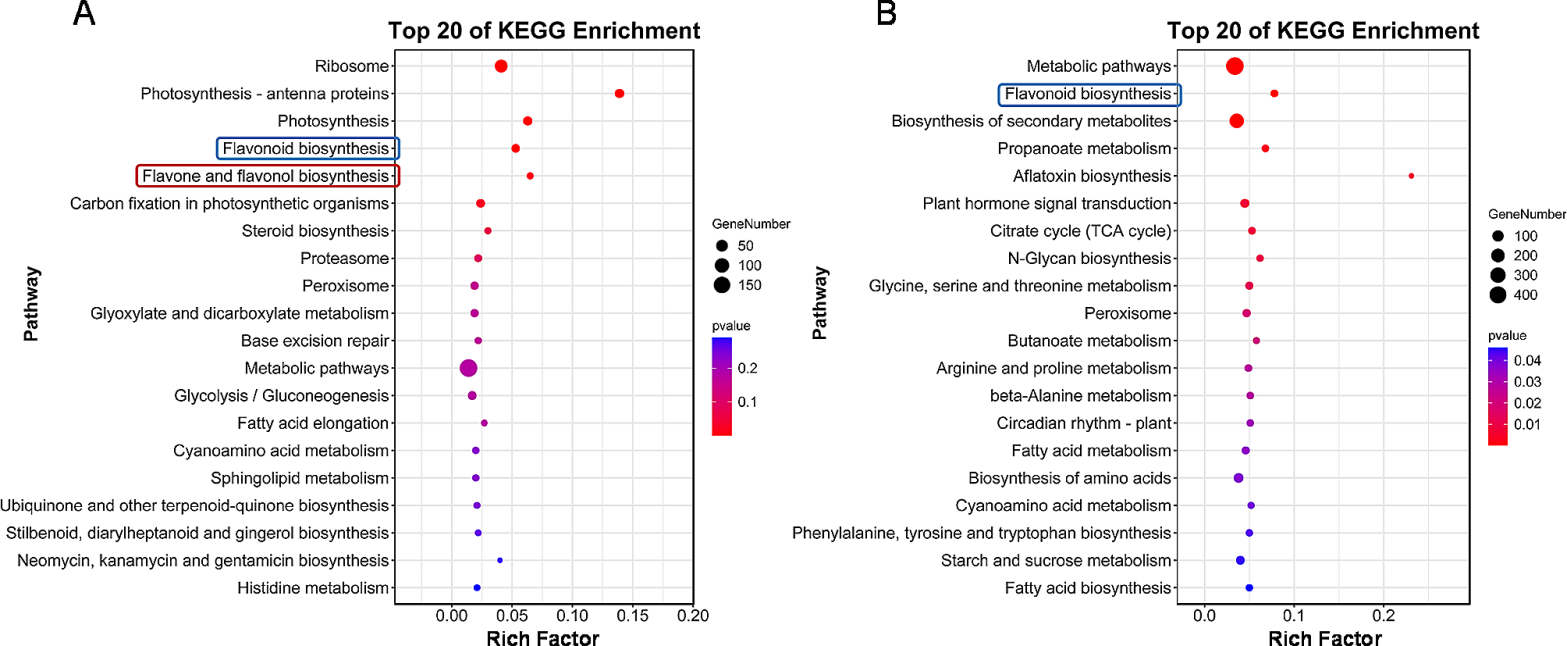
Bubble maps of top 20 KEGG-enriched pathways of genes in pink and turquoise modules. (**A**) pink module. (**B**) turquoise module. The x-axis and y-axis represent the rich factor and pathway name, respectively. The ‘Rich Factor’ refers to the ratio of genes in the target gene set that belong to a specific pathway, compared to all the genes present in that pathway within the background gene set. The image was created by the author, and KEGG copyright permission was obtained

As part of investigation into the potential functions of genes in each module, KEGG enrichment analysis was performed for genes within each module. The top 20 enriched pathways in each module were revealed that the genes from pink and turquoise modules were enriched in pathways related to flavonoid biosynthesis, such as “flavonoid biosynthesis”, “flavone and flavonol biosynthesis”, “biosynthesis of secondary metabolites”, and “metabolic pathways” (Fig. 4). Additionally, module-trait relationships indicated a positive correlation between K-3-glu and the turquoise module (*r* = 0.4, *p* = 5.1e*10^− 5^). Notably, numerous genes encoding key enzymes were found within these two modules, including but not limited to: *FLS*, *PAL*, *4CH*, *CHS*, *CHI*, *F3H*, and *UGT*. Due to the positive correlation between modules and flavonoid contents, as well as the results of KEGG pathway analysis, pink and turquoise modules were selected for further analysis.

### Identification of hub genes related to flavonoid biosynthesis

**Fig. 5:**
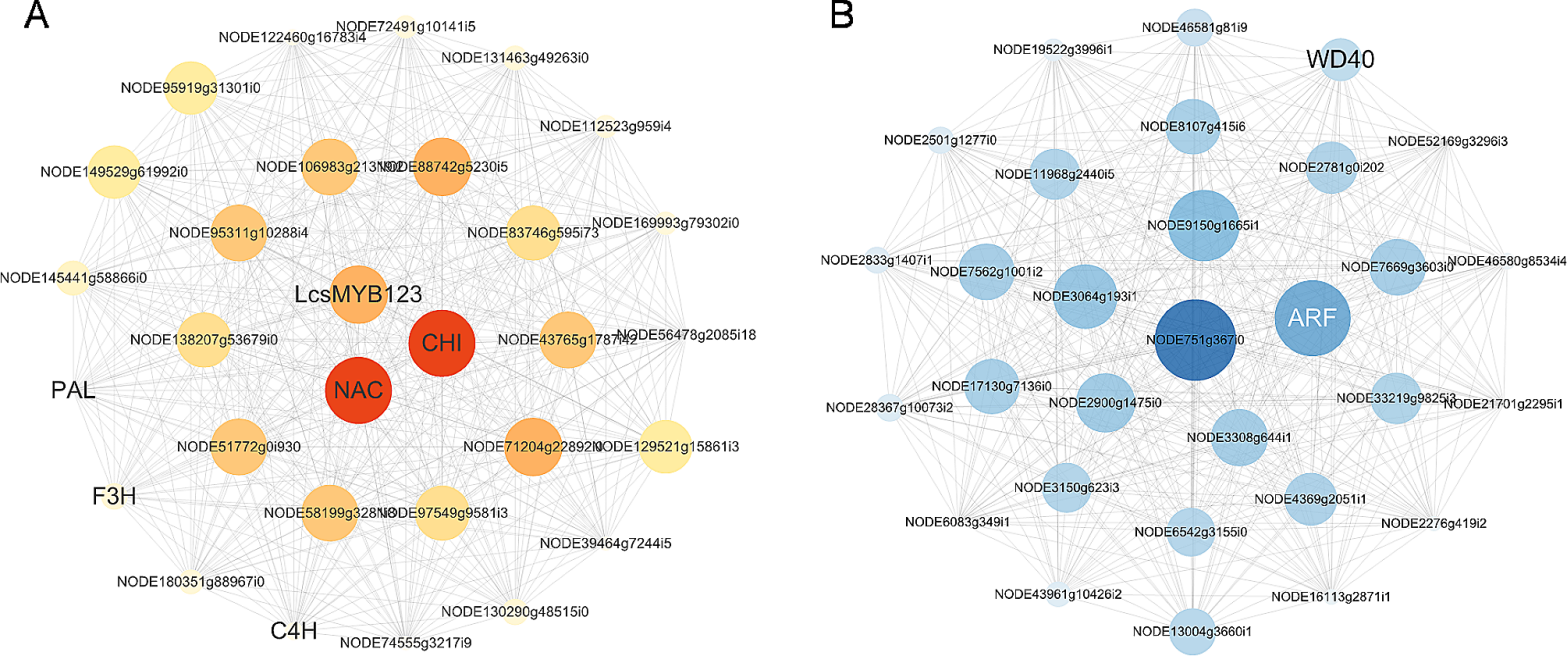
Hub gene visualization of the pink and turquoise modules. (**A**) pink module. (**B**) turquoise module. The larger the circle, the darker the color, and the greater the degree value

**Fig. 6:**
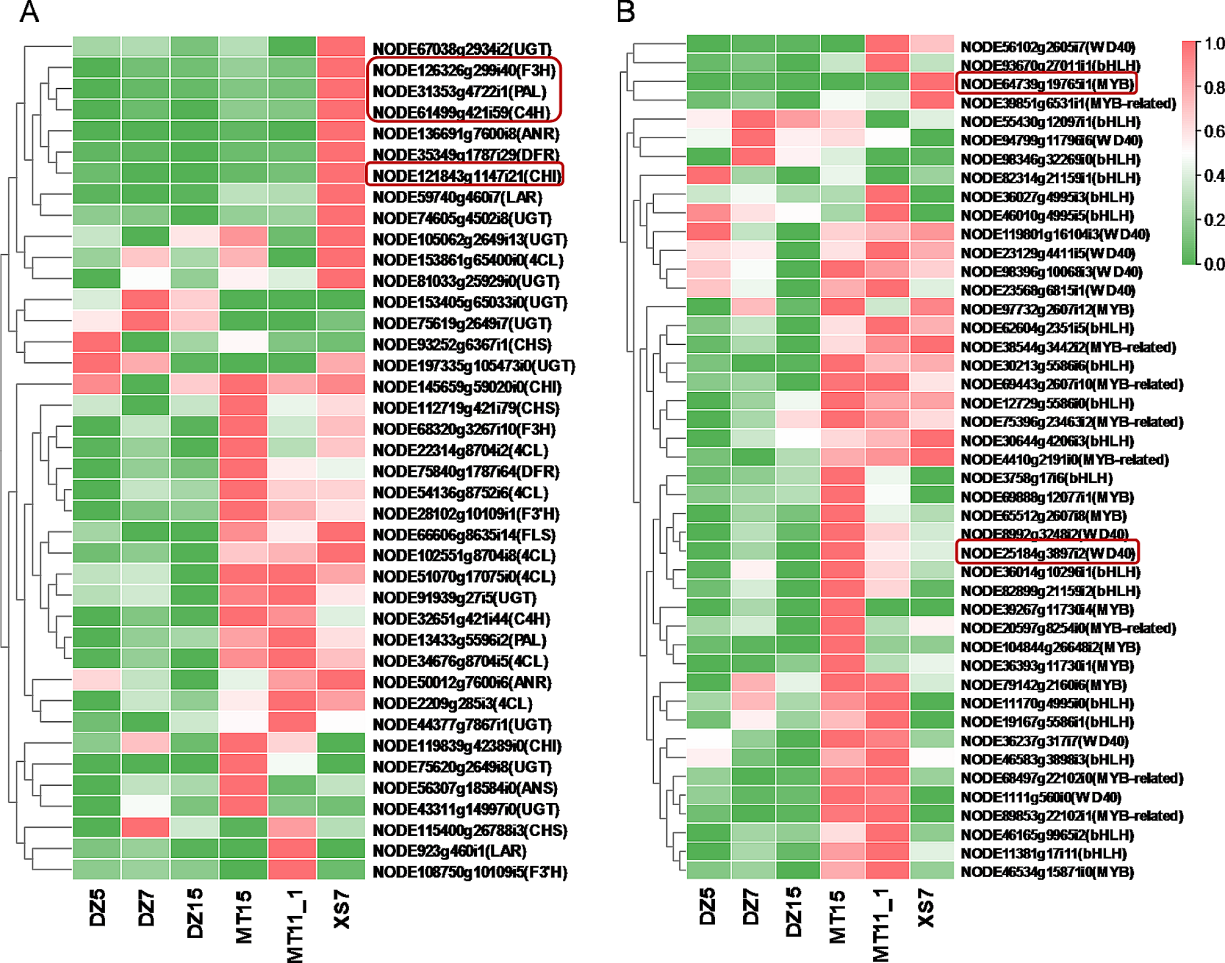
Expression patterns of genes involved in flavonoid biosynthesis pathways in pink and turquoise modules. (**A**) Structural genes expression levels. (**B**) TFs genes expression levels. The color scale from green to red represents the TPM values from low to high. The hub genes identified by WGCNA were represented in the round rectangular box. DZ5, DZ7, DZ15: the three samples with the lowest total amount of K-3-gal, Q-3-glu, Q-3-gal and K-3-glu. MT15, MT11_1, XS7: the three samples with the highest total amount of K-3-gal, Q-3-glu, Q-3-gal and K-3-glu

**Fig. 7:**
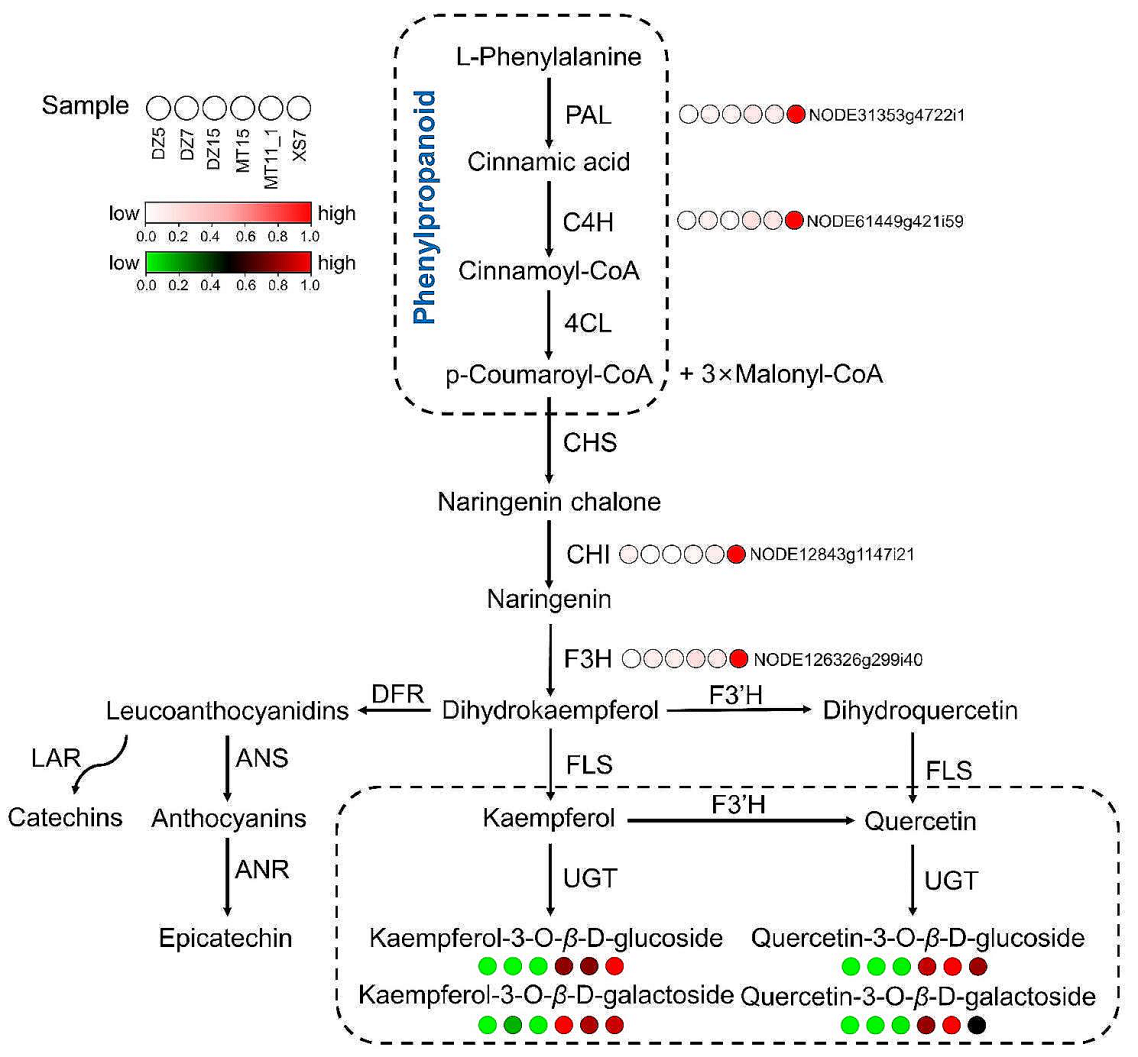
Pathway view heatmap of flavonoids and hub gene expressions in different *L. coreana* var. *sinensis* samples. Each sample is represented by a column, and each flavonoid/gene is represented by a row. Each flavonoid/gene expression profile is represented by a unique color, which accurately represents its relative abundance. Red indicates high-abundant flavonoids, whereas low-abundant flavonoids are presented in green. Red indicates high gene expression levels

In gene co-expression network, the top 30 genes with the highest degrees within the module were identified as hub genes. The enrichment analysis showed that genes within pink and turquoise modules were enriched in flavonoid biosynthesis, so the co-expression networks were constructed using Cytoscape. From the pink module, six genes *NODE64739g19765i1*, *NODE70694g17362i1*, *NODE12843g1147i21*, *NODE61449g421i59*, *NODE31353g4722i1*, and *NODE126326g299i40* were identified as transcription factor *MYB*, *NAC*, *CHI*, *C4H*, *PA*L, and *F3H*, respectively (Fig. 5A). In addition, one *ARF* (*NODE42009g6374i3*) and one *WD40* (*NODE25184g3897i2*) in turquoise module were also identified (Fig. 5B). Based on the gene expression data, structural genes and transcription factor genes (*MYB*, *bHLH, WD40*) related to flavonoid synthesis were analyzed within the pink and turquoise modules. Notably, high expression levels were found in samples with high flavonoid content (Fig. 6), which indicated that the genes within the pink and turquoise modules may have potential roles in the biosynthesis of flavonoids. In addition, the results showed a positive correlation between the gene expression levels of four structural genes identified by WGCNA and the corresponding flavonoid content, as evidenced by higher expression levels observed in six samples with high flavonoid content (Fig. 7).

**Fig. 8:**
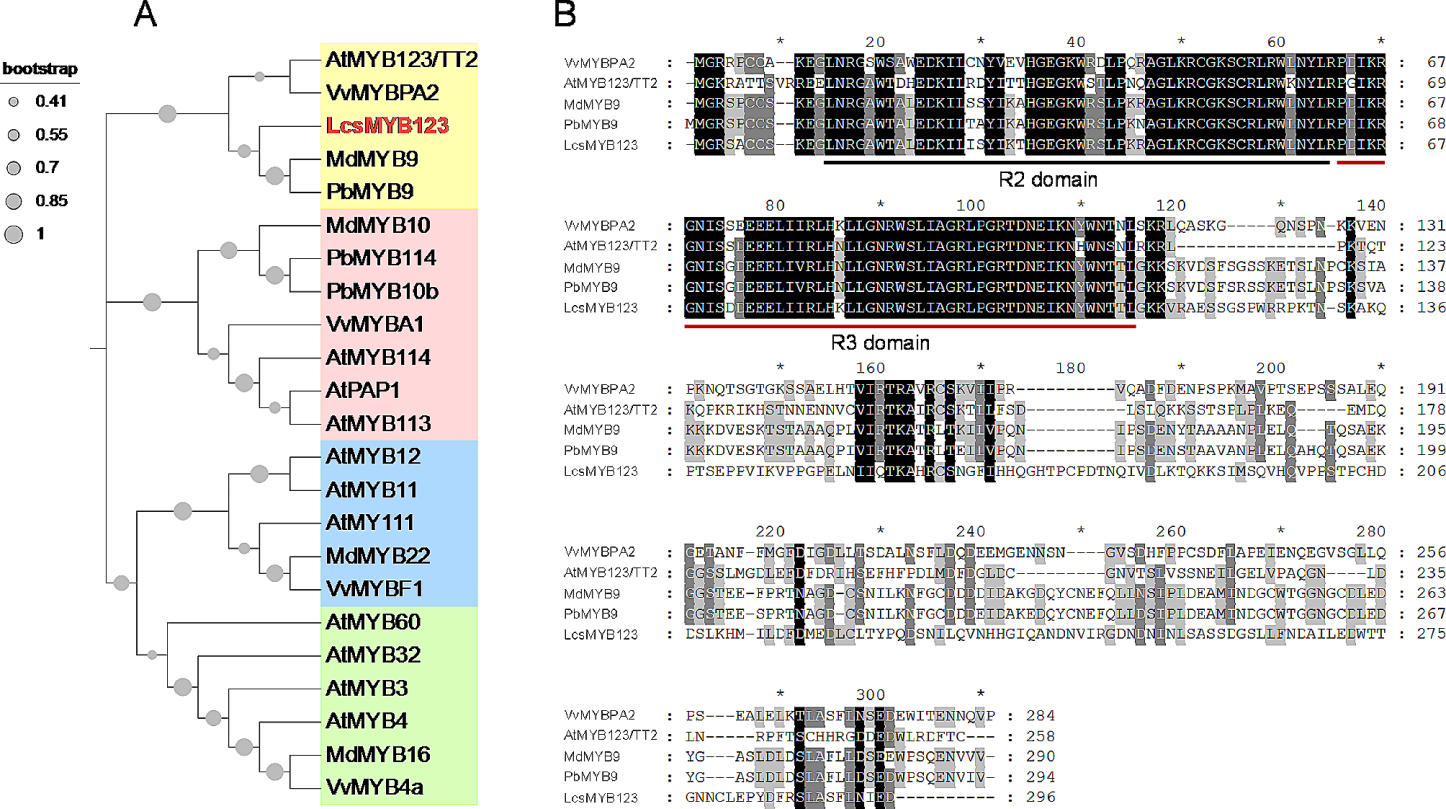
Phylogenetic analysis and protein sequence alignment of *LcsMYB123*. (**A**) Phylogenetic analysis of flavonoid-regulating MYBs in different plants. (**B**) Multi-alignment of the protein sequence of MYBs from different species. The R2 and R3 domains were underlined

The identified gene *NODE64739g19765i1* was named *LcsMYB123*. The LcsMYB123 protein contained two MYB DNA-binding domains, according to the conserved protein domain analysis. A multiple sequence alignment and phylogenetic tree of LcsMYB123 with other flavonoid-related R2R3-MYBs were generated to further define its roles. The phylogenetic analysis showed that LcsMYB123 had a higher sequence similarity to AtMYB123/TT2, PbMYB9 and MdMYB9 (Fig. 8).

### Validation of gene expression by qRT-PCR

**Fig. 9:**
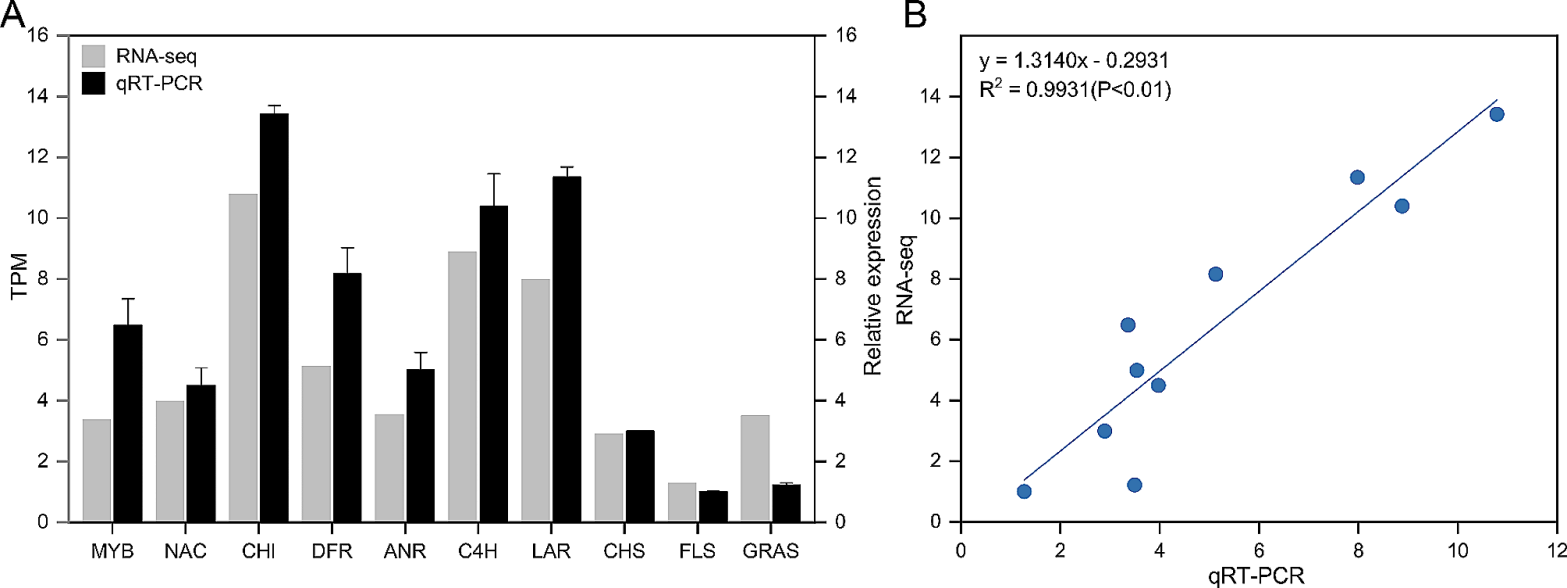
qRT-PCR verification. (**A**) Expression of 10 genes related to flavonoid biosynthesis. (**B**) Pearson’s correlation of gene expression between RNA-seq and qRT-PCR. The columns show RNA-seq expression (TPM) on the left y-axis, while lines represent qRT-PCR relative expression (2 ^−ΔΔCq^) on the right-axis

To validate the reliability of the transcriptome data, 10 genes related to flavonoid biosynthesis were selected for qRT-PCR verification (Fig. 9A). KEGG analysis revealed that the pink module was enriched in both flavonoid biosynthesis and flavone and flavonol biosynthesis. Since *L. coreana* var. *sinensis* mainly produce flavonols, genes in the pink module were chosen for verification. The average Cq values for the 10 genes were presented in Table S4. The correlation coefficient between RNA-seq and qRT-PCR results reached 0.9331 (*P* < 0.01) (Fig. 9B). It indicated that the transcriptome sequencing data was highly accurate.

## Discussion

### The main flavonoids in *L. coreana* var. *sinensis*

*L. coreana* var. *sinensis*, commonly known as the Eagle tea plant. Due to its high flavonoid content, Eagle tea has become one of the most popular traditional drinks in southwest of China, producing from buds or leaves of *L. coreana* var. *sinensis* [8]. Researches on flavonoids are beneficial to improve the development and utilization of this species. In contrast to traditional green tea, the major contituents in Eagle tea are flavonol glycosides, which have antioxidant, anti-inflammatory, and antineoplastic activities [53]. Yu et al. [54] first isolated and identified K-3-glu, and Q-3-glu in *L. coreana* var. *sinensis* and Chen et al. subsequently characterized Q-3-gal and K-3-gal [55]. Ma et al. further corroborated that K-3-gal and Q-3-gal were the dominant components in it [8]. However, K-3-glu and Q-3-glu were higher in content compared to the other two in this study. This could potentially be attributed to variations in environmental conditions at the sampling sites.

Environmental conditions are an important factor in determining the production of flavonoids [56]. They can be influenced by factors such as the growing region, climate, temperature, and light [57]. In this study, a total of 98 samples were collected from five different regions and each location provided more than fifteen samples for the research. The results revealed notable variations in flavonoid contents across diverse regions, with XS exhibiting significantly higher content compared to DZ. This could potentially be attributed to variances in climatic conditions, temperature patterns, soil composition, and precipitation levels among different regions.

### Sequencing and annotation

The secondary metabolism of *L. coreana* var. *sinensis* is poorly understood due to the lack of genomic data. It has been widely acknowledged that RNA-seq analysis is a valuable method for identifying genes involved in the metabolic processes in non-model species [58]. Full-length transcriptome sequencing is widely used for the identification of novel genes, as it provides more information and effectively addresses concerns regarding transcript redundancy and incomplete assembly in NGS technology. For example, Yang et al. identified 121,955 unigenes in *Panax notoginseng* [59]. Mi et al. identified 78,809 unique transcripts in *Camellia sinensis*, with 65,264 being complete coding sequences [60]. Based on the ONT and Illumina sequencing technologies, a total of 107,977 unigenes were identified and successfully annotated in seven public databases. The transcriptome sequences would serve as a great resource for gene expression studies, selective breeding, and cultivation of *L. coreana* var. *sinensis*, even related species in the Lauraceae family.

### Candidate structural genes involved in flavonoid biosynthesis

The weighted gene co-expression network analysis has been widely used in transcriptomics and metabolomics data to identify co-expression modules and candidate hub genes in plants [26]. In *Ginkho biloba*, WGCNA was performed to identified hub genes related to flavonoid accumulation [61]. Khan et al. identified five key enzyme-encoding genes (C*HI*, *F3’H*, *DFR*, *LAR* and *UFGT*) that controlled flavonoid biosynthesis [62]. We performed a weighted gene co-expression network analysis to identify key modules associated with flavonoid, resulting in the identification of 23 gene modules were obtained. Notably, the pink and turquoise modules exhibited significant enrichment in the flavonoid biosynthesis pathway. In the pink module, four structural genes (*CHI*, *C4H*, *PAL*, and *F3H*) that have been shown to play direct roles in the flavonoid pathway were screened [63–66].

The process of flavonoid biosynthesis initiates with the phenylpropanoid pathway [67]. In this pathway, phenylalanine is converted to p-coumaroyl-CoA catalyzed by PAL, C4H, and 4CL. Following this, CHS, the inaugural enzyme in the flavonoid biosynthesis pathway, facilitates the conversion of three molecules of malonyl-CoA and one molecule of 4-coumaroyl-CoA into naringenin chalcone. This naringenin chalcone is subsequently transformed into naringenin catalyzed by CHI [43]. F3H catalyzes the conversion of naringenin to dihydroflavonols, which are ultimately modified into kaempferol, quercetin, and myricetin by FLS, from their respective precursors, dihydrokaempferol, dihydroquercetin, and dihydromyricetin [18]. There exists an interaction between FLS and dihydroflavonol 4-reductase (DFR) resulting in a competitive pathway leading either to flavonol or anthocyanin biosynthesis [68]. Glycosylated flavonoids, which are prevalent natural compounds in plants, are derived from the conversion of flavonols to flavonol glycosides, a reaction catalyzed by UGTs [69]. The expression levels of several genes (*PAL*, *4CL*, *CHS*, *ANR*, *FLS*, and *LAR*) were significantly up-regulated, leading to an increase in flavonols in *Tetrastigma hemsleyanum* [70]. This finding suggested that *CHI*, *C4H*, *PA*L, and *F3H* may play crucial roles in the accumulation of flavonoids in *L. coreana* var. *sinensis.*

### Candidate transcription factors related to flavonoid biosynthesis

TFs, bind to cis-acting elements in target genes via their special gene binding regions to activate the expression of genes [71]. R2R3-MYBs are the most common and largest MYB TFs, playing a central role in regulating the transcriptional metabolism of flavonoid synthesis in plants [72]. Several members of the R2R3-MYBs have been recognized; however, none have been reported in *L. coreana* var. *sinensis* previously. TRANSPARENT TESTA 2 (TT2)-type MYBs are an R2R3-MYBs that have been widely reported as regulators in the PA pathway [29]. However, a positive regulatory relationship was found between *PpMYB123* and flavonols in nectarine (*Prunus persica*) [73]. Sun et al. cloned the gene *FtMYB31*, which is a homologous gene of *AtMYB123/TT2*, and demonstrated that it enhances the accumulation of rutin and total flavonols in transgenic tobacoo (*Nicotiana tabacum*) by upregulating the expression of *CHS*, *F3H*, and *FLS* [74]. Another study reported that *PbMYB9* stimulates flavonol production by binding to the *PbUFGT1* promoter [75]. Similarly, a hub gene named *LcsMYB123 (NODE64739g19765i1)* was identified in the pink module in the present study. Sequence analysis showed that *LcsMYB123* has a typical R2R3-conserved domain and phylogenetic analysis found it clustered with *AtMYB123/TT2*, *PbMYB9* and *MdMYB9*. It was believed that genes with similar sequences may have similar functions. It suggested that *LcsMYB123* is able to activate the flavonol pathway; however, more researches are needed to validate the functions of this gene.

Moreover, one *NAC* (*NODE70694g17362i1*) was identified in the pink module. NACs were found to enhance plant stress resistance through their modulation of flavonoid pathway*s* [76]. Another study suggested that *MdNAC9* may promote flavonol accumulation by activating *MdFLS* [77]. In the turquoise module, one *ARF* (*NODE42009g6374i3*) and one *WD40* (*NODE25184g3897i2*) were identified as hub genes. A study revealed that auxin may impact flavonol accumulation through the regulation of ARF protein on *CHS, CHI, FLS*, and *F3’H* [78]. In *Arabidopsis thaliana, ARF2* directly regulates the expression of *MYB12* and *FLS genes*, and indirectly regulates *MYB11* and *MYB111* genes increased flavonol content [22]. Additionally, it was discovered that a WDR gene (AN11) may control flavonol production with bHLH and R2R3-MYB proteins in *Entada phaseoloides* [79]. These results indicated that four TF genes screened in this study might influence flavonoid biosynthesis by modulating the expression of structural genes or TF genes. This study provides potential genes for future research and plays a crucial role in the breeding of high-flavonoid *L. coreana* var. *sinensis*.

## Conclusion

In our study, the first *de novo* hybrid transcriptome of *L. coreana* var. *sinensis* were sequenced using ONT and Illumina sequencing. A total of 126,977 unigenes were obtained, with 107,977 successfully annotated in seven public databases. Through a comprehensive transcriptome analysis combined with WGCNA, eight candidate genes influencing flavonoid biosynthesis were identified, including four structural genes and four TF genes. The transcription factor LcsMYB123 was suggested to have a potential role in the biosynthesis of flavonols in *L. coreana* var. *sinensis.* This research presents valuable insights into candidate genes that might be instrumental in the future genetic manipulation and improvement of *L. coreana* var. *sinensis*.

### Electronic supplementary material

Below is the link to the electronic supplementary material.

Supplementary Material 1

## Data Availability

The sequenced raw reads generated in this study have been submitted to the NCBI SRA database with BioProject: PRJNA992466.

## Abbreviations

ONT: Oxford Nanopore Technology
Q-3-gal: Quercetin-3-O-*β*-D-galactoside
Q-3-glu: Quercetin-3-O-*β*-D-glucoside
K-3-gal: Kaempferol-3-O-*β*-D-galactoside
K-3-glu: Kaempferol-3-O-*β*-D-glucoside
PAL: Phenylalanine ammonia-lyase
C4H: Cinnamate 4-hydroxylase
4CL: 4-coumarate: CoA ligase
CHS: Chalcone synthase
CHI: Chalcone isomerase
F3H: Flavanone 3-hydroxylase
F3’H: Flavonoid 3’-hydroxylase
FLS: Flavonol synthase
UGT: UDP-glucosyltransferase
SGS: Second-generation sequencing
TGS: Third-generation sequencing
PacBio: Pacific Bioscience
WGCNA: Weighted gene co-expression network analysis
TF: Transcription factor
HPLC: High-performance liquid chromatography
BUSCO: Benchmarking Universal Single-Copy Orthologs
