# Correction: Integrated transcriptomic and WGCNA analyses reveal candidate genes regulating mainly flavonoid biosynthesis in *Litsea coreana* var. *Sinensis*

**DOI:** 10.1186/s12870-024-05013-8

**Published:** 2024-04-25

**Authors:** Na Xie, Qiqiang Guo, Huie Li, Gangyi Yuan, Qin Gui, Yang Xiao, Mengyun Liao, Lan Yang

**Affiliations:** 1 Institute for Forest Resources and Environment of Guizhou, College of Forestry, Guizhou University, Guiyang, 550025 China; 2 College of Agriculture, Guizhou University, Guiyang, 550025 China

**Correction: BMC Plant Biol 24, 231 (2024)**

10.1186/s12870-024-04949-1

Following publication of the original article [1], the authors identified a minor typographical error in the author name of Qiqiang Guo. The correction is highlighted below in **bold**.

The incorrect author name is: Qiqaing Guo

The correct author name is: Qiq**ia**ng Guo

The author group has been updated above and the original article [1] has been corrected.

## References

[1] Xie N, Guo Q, Li H et al. Integrated transcriptomic and WGCNA analyses reveal candidate genes regulating mainly flavonoid biosynthesis in Litsea coreana var. sinensis. BMC Plant Biol 24, 231 (2024). 10.1186/s12870-024-04949-1. 10.1186/s12870-024-04949-1 PMC10985888 38561656

