# Supplementary material for: Integrated transcriptomic and WGCNA analyses reveal candidate genes regulating mainly flavonoid biosynthesis in Litsea coreana var. sinensis
Source: BMC Plant Biol. 2024 Apr 1;24:231. doi: 10.1186/s12870-024-04949-1 (PMC10985888; doi:10.1186/s12870-024-04949-1)
Supplement: Supplementary file 1 — Supplementary Material 1 [file 12870_2024_4949_MOESM1_ESM.docx]

Supplementary Materials

**Table S1** Primers used for the qRT-PCR assay.

| Gene symbol |  | Primer sequence (5' to 3') |
| --- | --- | --- |
| NODE64739g19765i1 (*MYB*) | Forward primer | CCACAAGCTCCTAGGGAACAG |
|  | Reverse primer | GAGTTGGTTTTGGGTCTTCGC |
| NODE70694g17362i1  (*NAC*) | Forward primer | TCCGGTTCCATCCCACTGAT |
|  | Reverse primer | TGCTCTGTTGGGTCGATTCC |
| NODE124009g10109i7  (*F3’H*) | Forward primer | ACGACTTCCGCCACATCC |
|  | Reverse primer | CCATCATCTCCACCACCATCT |
| NODE121843g1147i21  (*CHI*) | Forward primer | GGCACTGAGATGGTTATGGTTG |
|  | Reverse primer | TCTCCTGCTTTCCCCTTAAACT |
| NODE35349g1787i29  (*DFR*) | Forward primer | AGCCACAACAGTTAAGCGTG |
|  | Reverse primer | TTCTCCTTGGCAAAGTCCCA |
| NODE68908g7600i7  (*ANR*) | Forward primer | TGTCCCCAGTAGCACTTCTC |
|  | Reverse primer | GCAGCATATGTATCGCCCAG |
| NODE61499g421i59  (*C4H*) | Forward primer | GCGATTCAAGCTTCCTCCTG |
|  | Reverse primer | CCCCTGAGTGTGGAGAACTT |
| NODE59740g460i7  (*LAR*) | Forward primer | ATTCTCGACCAGCTCACCTTG |
|  | Reverse primer | CGAGAGGAGGAAGAACATCGG |
| NODE43762g421i48  (*CHS*) | Forward primer | CCAGAGCGAGTACCCTGATTAC |
|  | Reverse primer | ACTTCAACCACCACCATGTCTT |
| NODE66606g8635i14  (*FLS*) | Forward primer | AGGAGAAGGAGGAGTATGCGAT |
|  | Reverse primer | TTTGAGCACCCTTTCTTCCAGT |
| NODE27869g1717i6  (*GRAS*) | Forward primer | CGCCTTCTTCGACTCTACTCTC |
|  | Reverse primer | CTGAAATCGATGACATGGACGC |
| NODE57759g18792i1  (*18S rRNA*) | Forward primer | CAAAGTCGATGCTTGATGTTGC |
|  | Reverse primer | GCACCATCAATAACTCCAGGTC |

**Table S2** *Litsea coreana* var. *sinensis* unigenes annotated in public databases

| Databases | Number of annotated genes | Percentage(%) |
| --- | --- | --- |
| GO | 52,892 | 41.65 |
| KEGG | 34,899 | 27.48 |
| KOG | 61,557 | 48.48 |
| NR | 76,446 | 60.20 |
| Pfam | 96,438 | 75.95 |
| Swiss-Prot | 66,072 | 52.03 |
| TrEMBL | 53,978 | 42.51 |
| Total unigenes | 107,977 | 85.04 |

**Table S3** Sampling location and number.

| Sampling sites | Acronym | Sample number |
| --- | --- | --- |
| DaoZhen | ZA | DZ1 DZ2 DZ3 DZ4 DZ5 DZ6 DZ7 DZ8 DZ9 DZ10 DZ11 DZ12 DZ13 DZ14 DZ15 DZ16 DZ17 DZ18 DZ19 DZ20 |
| KaiYang | KY | KY1 KY3 KY4 KY5 KY6 KY7 KY8 KY10 KY11 KY12 KY13 KY14 KY15 KY16 KY17 KY18 KY19 KY20 KY21 KY22 KY23 KY24 KY25 |
| MeiTan | MT | MT1 MT2 MT3 MT5 MT6 MT7 MT8 MT9 MT10 MT11 MT12 MT13 MT14 MT15 MT16 MT17 MT18 MT19 MT6_1 MT8_1 MT9_1 MT10_1 MT11_1 |
| ZhenAn | ZA | ZA4 ZA6 ZA7 ZA8 ZA10 ZA11 ZA12 ZA13 ZA14 ZA15 ZA16 ZA18 ZA19 ZA20 ZA21 |
| XiShui | XS | XS1 XS2 XS3 XS4 XS5 XS6 XS7 XS8 XS9 XS10 XS11 XS12 XS13 XS17 XS20 XS21 XS22 |

**Table S4** The average Cq values of qRT-PCR genes.

| FLS | CHI | NAC | ANR | C4H | LAR | CHS | MYB | DFR | GRAS |
| --- | --- | --- | --- | --- | --- | --- | --- | --- | --- |
| 27.92 | 24.17 | 25.90 | 25.72 | 24.60 | 24.42 | 26.34 | 25.33 | 24.96 | 27.66 |

Supplementary Figures


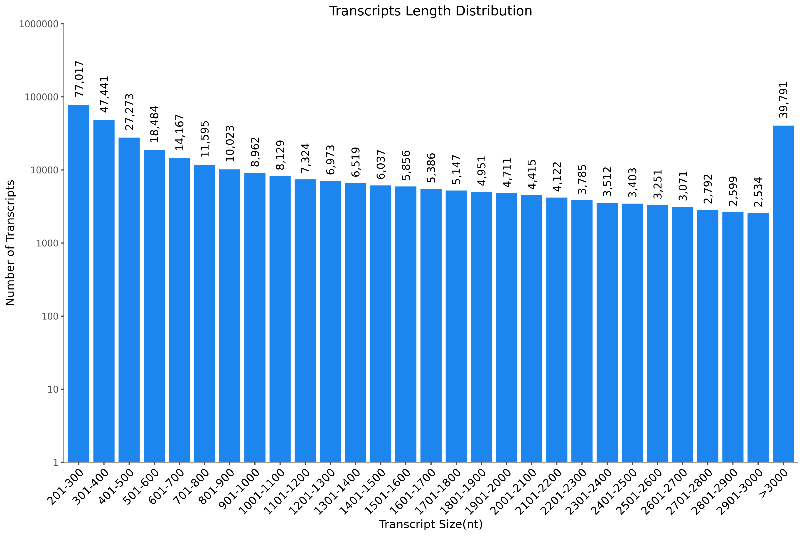


**Fig. S1** Length distribution of transcripts.





**Fig. S2** Gene Ontology (GO) classification of unigenes.

**Fig. S3** Number of unigenes of *L. coreana* var *sinensis* in each transcription factor families.


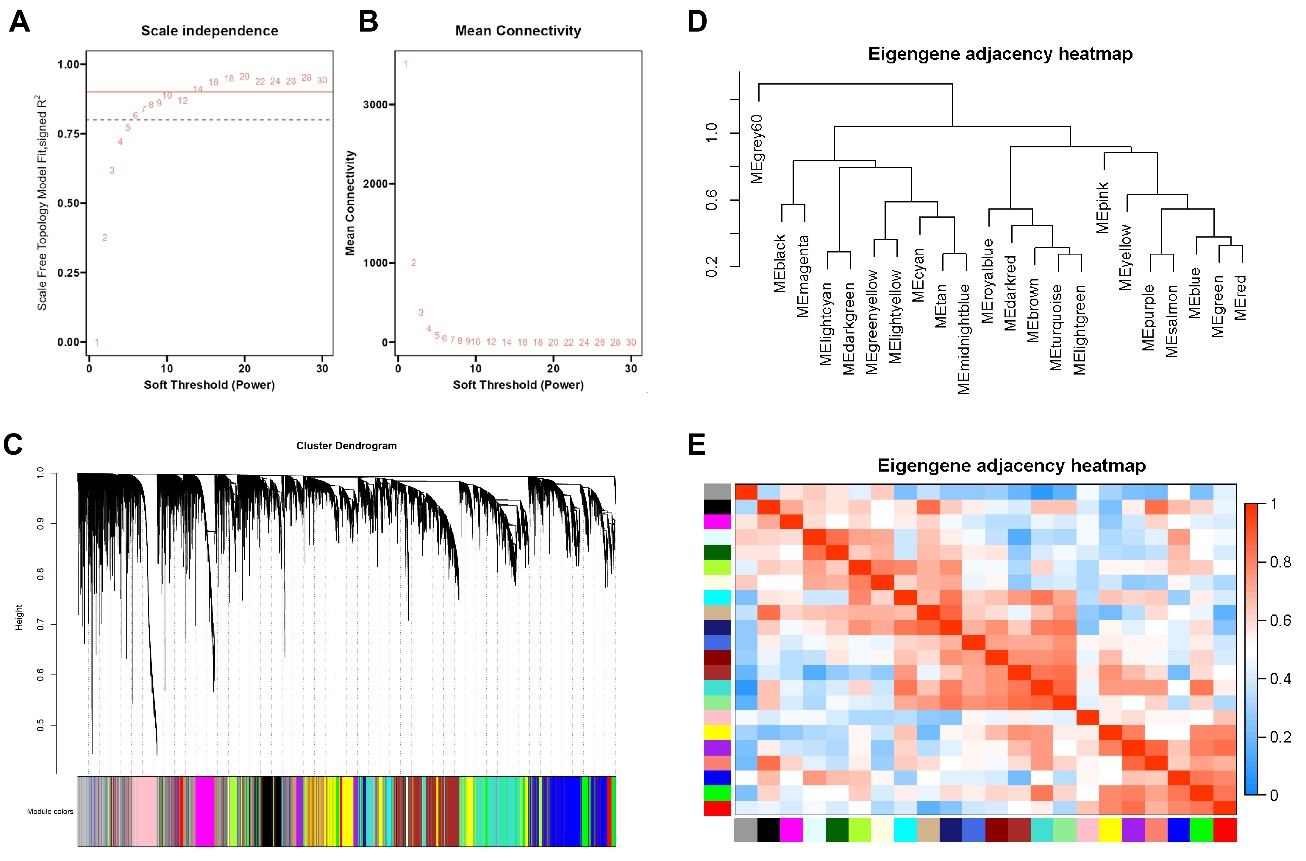


**Fig. S4** Construction of gene co-expression networks with WGCNA. A B Analysis of the network topology for various soft-thresholding (β value) powers. The blue dotted line is drawn at 0.8. C. Hierarchical clustering dendrogram of module eigengenes. D. Hierarchical clustering dendrogram of module eigengenes. E. Heatmap plot of the adjacencies in the eigengene network.
